# Fitness and Genomic Consequences of Chronic Exposure to Low Levels of Copper and Nickel in *Daphnia pulex* Mutation Accumulation Lines

**DOI:** 10.1534/g3.118.200797

**Published:** 2018-11-02

**Authors:** James K. Bull, Jullien M. Flynn, Frederic J. J. Chain, Melania E. Cristescu

**Affiliations:** * Department of Biology, McGill University, Quebec, Canada, H3A 1B1; † Molecular Biology and Genetics, Cornell University, New York, 14853; ‡ Department of Biological Sciences, University of Massachusetts, Lowell, Massachusetts, 01854

**Keywords:** genetic load, life-history evolution, mutational decay, mutation rate, metal pollution

## Abstract

In at least some unicellular organisms, mutation rates are temporarily raised upon exposure to environmental stress, potentially contributing to the evolutionary response to stress. Whether this is true for multicellular organisms, however, has received little attention. This study investigated the effects of chronic mild stress, in the form of low-level copper and nickel exposure, on mutational processes in *Daphnia pulex* using a combination of mutation accumulation, whole genome sequencing and life-history assays. After over 100 generations of mutation accumulation, we found no effects of metal exposure on the rates of single nucleotide mutations and of loss of heterozygosity events, the two mutation classes that occurred in sufficient numbers to allow statistical analysis. Similarly, rates of decline in fitness, as measured by intrinsic rate of population increase and of body size at first reproduction, were negligibly affected by metal exposure. We can reject the possibility that *Daphnia* were insufficiently stressed to invoke genetic responses as we have previously shown rates of large-scale deletions and duplications are elevated under metal exposure in this experiment. Overall, the mutation accumulation lines did not significantly depart from initial values for phenotypic traits measured, indicating the lineage used was broadly mutationally robust. Taken together, these results indicate that the mutagenic effects of chronic low-level exposure to these metals are restricted to certain mutation classes and that fitness consequences are likely minor and therefore unlikely to be relevant in determining the evolutionary responses of populations exposed to these stressors.

As the ultimate source of the genetic variation on which all other evolutionary processes act, novel mutations and the processes that generate them have received extensive theoretical and empirical attention. One key dimension of this research is the rate at which novel mutations occur. This parameter is relevant to understanding a range of biological processes including the evolution of sex (Kondrashov 1988), the adaptation of populations to novel conditions (Bell and Gonzalez 2009), and the fate of small populations (Lynch and Gabriel 1990). It is generally accepted that mutation rates vary both across and within species (Drake 2006). Among living organisms, species-specific per generation rates vary by at least four orders of magnitude with rates typically lower in species with larger effective population sizes (Lynch *et al.* 2016a). Within species, genotype-specific mutation rates have been shown to exist (Baer *et al.* 2005; Ness *et al.* 2015), determined in part by current mutation load (Sharp and Agrawal 2012; Sharp and Agrawal 2016). The extent to which these rates are affected by environmental factors or are fixed properties of specific genotypes has been a long-standing question in evolutionary biology (Plough 1917; Muller 1928; Klekowski 1976). Early studies focused largely on the effects of temperature and obtained mixed results (Muller 1928). Although studies since have shown that environmental effects on mutation rates are possible; *e.g.*, increased mutation rates at microsatellite loci in *Caenorhabditis* propagated at temperatures known to strongly reduce fitness (Matsuba *et al.* 2013) and increased mutation rates in mice exposed to airborne particulates associated with industrial activity (Somers *et al.* 2004), the circumstances under which they can occur and mechanisms involved remain largely unclear. This uncertainty is particularly true for multicellular organisms (but see Muller 1928; Zhong and Priest 2011; Matsuba *et al.* 2013; Sharp and Agrawal 2016), despite the extreme relevance of potential environmentally-driven modifications to mutation rates to the responses of wild populations to current anthropogenic environmental change.

In unicellular organisms, and particularly in prokaryotes (Bjedov *et al.* 2003), these questions are better studied (reviewed in Galhardo *et al.* 2007; Fitzgerald *et al.* 2017). Broadly speaking, mutation rates are often elevated in environments that are stressful to the organism in question (Galhardo *et al.* 2007). This appears to be true not only for stressors capable of damaging DNA directly or capable of interfering with the enzymatic processes that protect the genome (*e.g.*, metals, highly oxidizing conditions, Rose and Anderson 2005) but also for more general stressors, such as carbon starvation or elevated temperatures (Ogur *et al.* 1960; Bjedov *et al.* 2003). Increased mutation rates under general stressors often involve the “SOS response”, whereby stress promotes the preferential use of error-prone polymerases and suppression of some elements of usual DNA monitoring and repair pathways (Maclean *et al.* 2013). The increased mutation rate under the SOS response, or via other pathways under stress, has been termed stress-induced mutagenesis (SIM) and proposed explanations range from neutral to adaptive (Echols 1981; Lynch 2010; Lynch *et al.* 2016a), with theory and experimental data indicating the relative importance of each explanation is likely context and system dependent (reviewed in Maclean *et al.* 2013). In addition to increased rates under stress, the spectrum of produced mutations can be modified. For example, the fraction of GC-producing single nucleotide mutations (SNMs) increased with increasingly acidic conditions in a coral reef pathogen (Strauss *et al.* 2017), while this fraction decreased with increased exposure to an antibiotic in *Escherichia coli* (Long *et al.* 2016).

Despite the substantial body of knowledge in unicellular organisms, multicellular organisms might be expected to behave differently with regards to mutational responses to stress for at least three reasons. First, under benign conditions mutation rates for multicellular organisms are typically orders of magnitude higher than for unicellular organisms (Lynch *et al.* 2016a), plausibly driven largely by less efficient downward selection on the mutation rate in smaller populations (Lynch 2010). Since mutation load is proportional to mutation rate and thus greater in multicellular organisms (Haldane 1937; Kimura and Maruyama 1966), temporary increases in the mutation rate may be proportionally more harmful in multicellular organism, more strongly favoring factors that stabilize the rate in the face of stress (Baer 2008). Second, few multicellular organisms produce sufficient numbers of offspring that the increased chance of rare adaptive mutations due to a greater total number of mutations outweighs the decreased average fitness of other offspring (Lynch 2007). Third, the distinction between the germline and soma in many multicellular organisms means only mutations in a subset of cells could potentially confer evolutionary advantages, while all cells bear the burden of the typically negative effects of additional mutations (Fitzgerald *et al.* 2017).

Empirically, our knowledge of mutation under stress in multicellular organisms is currently limited to a few studies and organisms (*Drosophila*, Muller 1928; Agrawal and Wang 2008; Sharp and Agrawal 2012; Sharp and Agrawal 2016; *Caenorhabditis*, Matsuba *et al.* 2013; *Arabidopsis*, Mackenzie *et al.* 2005; Jiang *et al.* 2014) and these largely support the premise of increased mutation rates under stress. Importantly, stressors in these studies are not limited to heavy, acute exposures to known mutagenic compounds (*e.g.*, petroleum products, Klekowski *et al.* 1994; industrial pollutants, Arutyunyan *et al.* 1999) but include relatively mild but sustained stresses (*e.g.*, elevated temperatures in *C. elegans* in Matsuba *et al.* 2013). Within these studies, the only two studies that examined the mutation spectra at the molecular level across the whole genome (Jiang *et al.* 2014; Sharp and Agrawal 2016) give partially inconsistent results. For example, Jiang *et al.* (2014) found a twofold increase in SNM rates under salinity stress for *A. thaliana*, while Sharp and Agrawal (2016) found no effect on SNM rates of high- *vs.* low-quality genetic backgrounds in *D. melanogaster*. Given that these studies used different organisms and stressors, variable results are hardly surprising and additional studies are needed to infer generalities. Understanding effects of stressors on the mutation spectra across the genome is important given that different classes of mutations (*e.g.*, SNMs *vs.* INDELs) may have different fitness consequences in natural populations (Keith *et al.* 2016; Flynn *et al.* 2017), potentially driving different evolutionary outcomes. For example, if INDELs are on average more deleterious than SNMs, due to their ability to cause frameshift mutations (Sung *et al.* 2016), stressors that result in predominantly more INDELs could theoretically increase chances of rapid mutational decline more so than stressors resulting in predominantly more SNMs (Haldane 1937).

As highlighted above, given that mutations affect populations through their fitness effects, considerations of the effects of environmental stress on mutation rates must include whether the distribution of fitness effects of mutations is also affected. Fitness effects of novel mutations range from deleterious to advantageous (Halligan and Keightley 2009), with the fitness effects of the majority of mutations following a continuous distribution peaking at slightly deleterious, and a separate smaller class of strongly deleterious mutations involving loss of vital functions (Davies *et al.* 1999; Keightley and Lynch 2003). Some research indicates there may also be a moderate fraction of substantially beneficial mutations (Shaw *et al.* 2000). While strongly deleterious mutations, such as those causing death or sterility, are likely rapidly removed by natural selection, moderately deleterious mutations may become fixed in populations when they have small enough fitness-effects for their dynamics to be largely dominated by genetic drift (Lynch and Gabriel 1990). Thus, the extent to which modified mutation rates under stress present a threat or benefit to natural populations should depend on the distribution of “additional” mutations incurred (Kimura 1967).

To investigate if chronic stress is mutagenic in a multicellular organism, the water flea *Daphnia pulex*, we conducted a mutation accumulation (MA) experiment in which lines were propagated in the presence of low but environmentally relevant concentrations of copper and nickel, or under metal-free control conditions with and without selection. Mutation accumulation is an experimental design which minimizes the effects of selective forces and allows mutations to accumulate in a near-neutral manner (reviewed extensively in Halligan and Keightley 2009). This is achieved by subjecting initially identical lines to single-individual (for asexually producing or selfing species) or single-pair (for typically sexually reproducing species) bottlenecks, ideally on a per-generation basis (Muller 1928). Copper and nickel were chosen as stressors as both life-history experiments and gene expression studies have shown acute exposure to these metals can be stressful to *Daphnia pulex* (Asselman *et al.* 2012). Further, *in vitro* interactions between these metals and DNA can lead to a range of types of DNA damage including double-stranded breaks and nucleotide modifications (Beyersmann and Hartwig 2008), which when incorrectly repaired can lead to novel mutations (Lieber 2010). The extent to which these acute effects under high concentrations occur under chronic low-level exposure, such as used here, is unclear. Specifically, we test if lines exposed to copper and/or nickel show more rapid declines in fitness, measured as intrinsic rate of increase and as body size, and/or greater numbers of SNMs, multinucleotide mutations (MNMs), small (<50 bp) INDELs, and/or loss-of-heterozygosity (LOH) events. This was achieved by measuring a range of life history traits using a classical life-history experiment and by sequencing the whole genomes of a subset of metal-exposed and control MA lines after more than 100 generations of mutation accumulation.

## Materials and Methods

### Mutation accumulation lines and the non-MA population

The experiments described here used *Daphnia pulex* mutation lines from a long-term mutation-accumulation (MA) experiment that had been running for over 5 years, or more than 120 generations on average. Briefly, a single female *D. pulex* was sampled from Canard Pond, Ontario, Canada, and cultured parthenogenetically to provide sufficient descendants to initiate 200 single-individual mutation accumulation lines. Fifty lines were assigned to each of the following treatments (herein “metal treatments”); control (cultivated in standard medium, Celis-Salgado *et al.* 2008), Ni (standard medium plus 80 μg/L nickel), Cu (standard medium plus 40 μg/L copper), Ni+Cu (standard medium plus 80 μg/L nickel and 40 μg/L copper). These metal concentrations were chosen for two reasons. First, they are representative of existing levels of nickel and copper pollution in historically polluted lakes in the Sudbury region, Ontario, Canada where *D. pulex* is present (Keller and Yan 1991; Celis-Salgado *et al.* 2016). Second, preliminary 14-day toxicity tests indicated that these concentrations have no detectable effects on survival or reproduction in this lineage in the medium used (unpublished data). Thus, these concentrations allow us to study effects of chronic mild stress. All MA lines were maintained in 50 mL vials with 20 ml of medium. Selective forces were minimized within lines by enforcing single-individual-bottlenecks each generation. This was achieved by transferring a single haphazardly selected neonate per line to fresh medium approximately every 11-13 days. This transfer interval was chosen to balance the needs of advancing generation number (favoring early transfer schedules) and allowing the majority of lines to produce at least a single clutch and maintaining generation synchronicity (favoring late transfer schedules), based on the typical reproductive schedule of this lineage; transfer-to-transfer timing variability was due to logistic constraints. Individuals that failed to reproduce before a transfer were transferred to fresh medium for up to two transfers before being declared sterile (termed carry-overs). Up to two previous generations were maintained as backups in case of death or sterility of the individual selected. The number of generations of each MA line was counted and kept track of at each transfer. Although using backups increases the effective population size and allows some selection to occur, backups use was infrequent (4.8% of all transfers) and unavoidable for the continuation of the experiment. Where backups were employed, a sibling of the individual being replaced was chosen haphazardly where possible, or if this was not possible, a sibling of that individual’s mother. In rare cases, multiple generations may have existed in the backup, thus it is possible we accidentally replaced an individual with the offspring of a sibling rather than the target sibling. If this occurred, it would result in underestimating the number of generations passed. The potential effects of such underestimation and uncertainty in generation number on our results, as well as potential effects of variability in generation-times within lines due to carry-overs, are explored in depth in Supplementary Text 1, but do not qualitatively affect the outcomes presented here.

All lines were fed twice weekly with a green algae mixture containing *Ankistrodesmus sp*., *Scenedesmus sp*. and *Pseudokirchneriella sp*. and were kept in culture chambers at a constant 18° and 70% humidity, and under a 12h: 12h, light: dark cycle. At the same time as the MA lines were established, a single large (N ≈ 100 - 250) population was established from descendants of the same progenitor in a 15 L tank (herein “non-MA population”). No bottlenecking was performed in this population, and thus selection was allowed to act. Under the assumption that the majority of mutations are deleterious (Eyre-Walker and Keightley 2007) and removed by selection, individuals in this population should retain phenotypes similar to the founding mother and are therefore an appropriate control against which to measure fitness changes under MA (Katju *et al.* 2015). Since our experiment was founded using a wild-caught individual, rather than a long-term laboratory population well-adapted to culture conditions, it is likely a subset of mutations advantageous under these conditions were positively selected. This assumption-violation should affect the absolute rates of change observed but not the comparison between treatments, which is our main interest. Further, previous genetic analysis of the non-MA population found no evidence for selective sweeps, indicating any violation is unlike to be major (Flynn *et al.* 2017). The non-MA population was fed twice weekly with the same algae mix as the MA lines and subjected to the same environmental conditions as the control MA lines.

### Life-history assays – Experiment

To assess the fitness consequences of mutations that arose during the MA experiment, the following were assessed for each of 40 lines after an average of 131.5 ± 7.9 (mean ± SE) generations of propagation (Table 1); body size at first clutch release, age at release for the first four clutches, number of progeny per clutch for the first four clutches, and longevity. These lines were composed of 10 control lines, 10 Cu lines, 10 Ni+Cu lines and 10 individuals from the non-MA population; no Ni lines were assessed due to logistical constraints. Where possible, lines were selected to overlap with those used in the genomic analyses described below (87%), with additional lines randomly chosen (Table S1). The traits were chosen to represent a range of life-history traits that affect the fitness of *Daphnia* in the wild and for comparability to previous studies (*e.g.*, Lynch 1985; Lynch 1989; Latta *et al.* 2013). Generation number differed slightly but significantly across treatments (Table 1, one-way ANOVA, F_2,27_ = 4.00, *P* = 0.03; TukeyHSD, control lines *vs.* Cu lines *P* = 0.02, other comparisons non-significant). This difference in propagation rate was unavoidable due to evolved differences in reproductive schedule between treatments over the course of the MA experiment (see results). To account for this, per generation estimates of fitness trait changes (and below, of genomic mutation rates) were used rather than absolute differences. Life-history assays followed standard protocols (Lynch 1985), and were performed under the same environmental conditions as for the propagation of the control MA lines. In these assays, up to seven sublines were established within each line for trait measurement. Details are provided in Supplementary Text 2. At the time of establishment of the life-history assays, 43 of 50 control MA lines, 47 of 50 Cu MA lines, and 49 of 50 Ni+Cu MA lines were extant.

**Table 1:** Mean ± SE (range) number of generations of mutation accumulation (MA), trait value, mutational bias (ΔM), coefficient of mutational variation (CV*_m_*), and mutational heritability (V_m_/V_E_) for intrinsic rate of increase (r) and body size for *Daphnia pulex* mutation-accumulation (MA) lines exposed to metal-free conditions (Control) and to low-levels of copper (Cu) or nickel and copper (Ni+Cu), and for a non-MA population (see text). ΔM is calculated relative to the non-MA population. n = 10 per treatment. ‘gen’ = generation throughout

Treatment	Non-MA population	Control	Cu	Ni+Cu
MA generations	N/A	126.7 ± 11.0	135.7 ± 4.2	132.2 ± 4.1
**r**				
- (unitless)	1.30 ± 0.03	1.29 ± 0.04	1.28 ± 0.07	1.23 ± 0.11
	(1.24 - 1.32)	(1.19 - 1.33)	(1.14 - 1.33)	(1.02 - 1.32)
- ΔM (%/gen * 10^2^)	N/A	−0.01 ± 0.79	−1.32 ± 1.13	−1.03 ± 1.33
- CV*_m_* (%)	N/A	6.01	6.39	8.17
- ΔCV*_m_* (%/gen)	N/A	0.047	0.047	0.062
- V_M_/V_E_	N/A	0.018	0.009	0.034
**Body size**				
- (mm)	2.46 ± 0.06	2.41 ± 0.10	2.46 ± 0.10	2.40 ± 0.14
	(2.31 - 2.52)	(2.17 - 2.50)	(2.22 - 2.58)	(2.13 - 2.55)
- ΔM (%/gen * 10^2^)	N/A	−1.95 ± 1.01	0.83 ± 0.57	−1.05 ± 1.47
- CV*_m_* (%)	N/A	10.80	5.23	12.00
- ΔCV*_m_* (%/gen)	N/A	0.085	0.039	0.091
- V_M_/V_E_	N/A	0.022	0.010	0.067

### Life-history assays - Statistical analyses

Initial analyses revealed strong correlations among measured traits within lines (Table S2). To summarize across correlated traits in a biologically meaningful way, we determined intrinsic rate of increase (r) for each line by calculating partial life-history tables for each line. We then used simulations to determine r from these tables; details are provided in Supplementary Text 3. This and all further analyses, except where noted, were performed using R v3.4 (R Core Team 2015). We quantified the effects of mutation on r and body size as mutational bias (ΔM), the per generation change in mean trait value due to mutation, and as the coefficient of mutational variation (CV*_m_*), a standardized measure of the increase of among-line variance in trait value due to each line containing unique sets of mutations (Mukai 1964).

We determined mutational bias (ΔM) for r and for body size by using weighted least squares regression. For each trait, this was repeated using all MA lines together and for each treatment (Control, Cu, Ni+Cu) separately. For all regressions, per line trait value (r) or mean (size) was a function of number of generations of MA, with the influence of observations weighted inversely to the standard deviation of trait values of sublines within a particular MA line. To ensure that these analyses reflect changes from the initial phenotype, regressions were forced to pass through, at generation zero, the mean of trait values of the 10 non-MA population lines. This mean was weighted as above. Since our simulations provide only one estimate or r per line, r was recalculated on a per-subline basis excluding sublines that died before life-history data were collected and the per-line standard deviation of these values were used as weights during regressions. The slopes of these regressions are mutational bias and were standardized as per generation percent changes of initial values. Additionally, to test if ΔM differed between treatments for either r or body size, we repeated the above analyses for all MA lines together but included treatment as a fixed factor, and tested the interaction between treatment and generation using ANOVA. To verify the appropriateness of this approach, we used regression to test if per-line rate of change in r and in body size was linearly related to generation number. For this test, per line rates were calculated as the mean value of that line minus the mean of non-MA population lines, divided by number of generations of MA for that line.

We calculated the coefficient of mutational variation (CV*_m_*) for r and for body size using one-way ANOVA to partition variance among sublines values into between line (mutational) and within line (environmental) variance. The square root of mutational mean square variance was then divided by the mean trait value for that treatment and expressed as a percent to obtain CV_m_ (Houle *et al.* 1996). Since mean generation number differs between treatments, we also express accumulation of CV*_m_* on a per generation basis (ΔCV_m_). For r and for body size, this was repeated using all lines together and for each treatment separately. Similarly, we calculated mutational heritability using all lines together and for each treatment separately following Houle *et al.* (1996). As an additional test of whether lines within a treatment had diverged over the course of the MA experiment, we performed single-factor ANOVAs for r and for body size as a function of line within each treatment. To explicitly test if variance differed between treatments for either r or body size, we also preformed Bartlett-tests. To estimate the extent to which uncontrolled microenvironmental variation may have affected our results, we calculated broad-sense heritability for r and for body size following Griffiths *et al.* (2015).

### Genomic analyses – Sequencing

To examine if metal exposure resulted in more sequence-level mutations, we sequenced lines from metal and control treatments and analyzed resultant reads to identify single-nucleotide mutations (SNMs), multiple-nucleotide mutations (MNMs, multiple SNMs within a 50bp stretch), small (< 50 bp) insertion-deletion events (INDELs), and loss-of-heterozygosity events (LOH) that had arisen during mutation accumulation. We examined mutations in the nuclear genome only. Large-scale (> 500 bp) deletions and duplications in these lines have been estimated elsewhere (Chain *et al.* 2018). Specifically, 28 control lines, 9 Ni lines, 9 Cu lines and 9 Ni+Cu lines (n = 55 total) were sequenced using 100bp or 125bp paired-end reads on Illumina HiSeq 2000 and 2500 platforms. Ten sequencing lanes were used across 4 runs. Within treatments, lines were randomly selected (Table S1). Control lines had been propagated for 82.3 ± 4.7 generations on average. Other lines were harvested later in the MA experiment and had been propagated for 120.5 ± 10.4 generations on average. A subset of the genomes were used in previous studies, including 24 of the control MA lines (Flynn *et al.* 2017) and all the metal lines (Chain *et al.* 2018). For each line, 5-10 clonal offspring were collected, treated with antibiotics to reduce potential endosymbiont and parasite loads and fed sterile beads to clear gut contents (Fields *et al.* 2015). DNA was extracted from pooled individuals using a modified CTAB protocol (Doyle and Doyle 1987) and quantified with PicoGreen Quant-IT (Invitrogen). Library preparation was performed using a modified version of the Illumina Nextera protocol (Baym *et al.* 2015). Sequencing was performed by the Genome Quebec Innovation Centre at McGill University. At their relevant times of sequencing, 50 of 50 control MA lines, 47 of 50 Ni MA lines, 47 of 50 Cu MA lines, and 49 of 50 Ni+Cu MA lines were extant.

### Genomic analyses - Identification of mutations

Germ-line mutations that had arisen during mutation accumulation were identified using a combination of standard bioinformatic approaches and filtering using custom scripts. Briefly, reads were preprocessed with SeqPrep (https://github.com/jstjohn/SeqPrep), aligned to the reference *Daphnia* genome (Colbourne *et al.* 2011) using BWA v0.7.10 (Li and Durbin 2009) and filtered using a combination of Picard tools v1.123 (https://broadinstitute.github.io/picard/) and GATK v.3.3.0 (Mckenna *et al.* 2010). Base and variant calling was then performed in GATK v.3.3.0, and sites containing putative variants were subjected to custom binomial tests and filtering to ensure patterns across all MA lines were consistent with true mutations (Flynn *et al.* 2017; full details are provided in Supplementary Text 4). Although a more recent genome assembly exists for *D. pulex* (Ye *et al.* 2017), we used the Colbourne *et al.* (2011) assembly to allow comparison with previous papers reporting findings from these MA lines (Flynn *et al.* 2017; Chain *et al.* 2018), and to other *Daphnia* studies (Lynch *et al.* 2016b). Due to our strict filtering criteria, it is extremely unlikely somatic mutations are captured in these analyses as they will only be present in a subset of cells in a single individual in the pooled DNA extraction and will therefore not conform to expected patterns of allele coverage. This approach allowed us to focus on germ-line mutations, which due to their heritable nature can affect long-term evolutionary processes.

Sites that mutated from a heterozygous (‘Het’) to homozygous (‘Hom’) state (‘Het-Hom’ sites) represent a special case as they are more likely due to gene-conversion processes or hemizygous deletions that effect multiple sites (*i.e.*, LOH events) than a result of SNMs (Omilian *et al.* 2006). Based on our observed SNM mutation rate at homozygous sites, assuming no difference in mutation rate between homozygous and heterozygous sites, the fraction of the genome recovered, and a 0.7% per-site heterozygosity in the ancestor (Flynn *et al.* 2017), we would expect to observe only one true Het-Hom SNM across the recovered genomes of all sequenced MA line. Therefore, we only considered Het-Hom sites if they were part of putative LOH events. For an LOH event to be called, we required at least two inferred Het-Hom sites covering at least 200 bp and no evidence of heterozygosity at sites within the affected stretch as verified manually using the Integrative Genomics Viewer (Robinson *et al.* 2011). We chose a minimum distance of 200 bp based off preliminary validations that showed LOH events below this size could not reliably be validated (10 of 11 putative LOH events < 200 bp validated were false positives, Table S3). As detection of LOH relies on Het-Hom sites, we cannot detect adjacent ancestrally-homozygous sites affected by the causal event, *i.e.*, event sizes are minimum estimates. To address this, we estimated maximum event size as the distance between unaffected Het sites bounding the LOH event and used the average of minimum and maximum event sizes in further calculations. In several cases, MA lines had multiple scaffolds that were completely homozygous, and where previous information indicated such scaffolds belonged to the same chromosome (Colbourne *et al.* 2011; Xu *et al.* 2015) or where patterns of LOH across multiple lines indicated they were likely on the same chromosome; these were assigned to the same LOH event. For multi-scaffold LOH events, we cannot estimate maximum event size as relative scaffold order and orientation are unknown and we used the minimum size estimate in further calculations. All such events were large (>700,000 bp) and this underestimation should be negligible. Due to the very large size of some identified LOH events (see results) we allowed putative LOH events to overlap among lines. To differentiate between LOH caused by gene-conversion-like events and hemizygous deletions, we used samtools (Li *et al.* 2009) to calculate a measure of standardized relative read coverage for each event. For this, we divided the average coverage across the LOH event in the affected line by that averaged across that region for all lines. We then corrected this value for the difference in genome-wide coverage for that line compared to average genome-wide coverage across all lines. LOH events with standardized relative coverage≥ 0.75 were inferred to be caused by gene-conversion-like events and those with standardized relative coverage< 0.75 were inferred to be caused by hemizygous deletions. For multi-scaffold LOH events, we calculated this statistic for the largest scaffold affected.

### Genomic analyses - Validations

Given the large potential for false-positives when using whole genome sequencing data to detect novel mutations (Olson *et al.* 2015), we validated a subset of detected mutations to ensure our pipeline provided reliable calls. A total of 26 SNMs, 5 INDELs and 12 LOH events were randomly selected for validation by Sanger sequencing. For each putative mutation we used Primer3 (Untergasser *et al.* 2012) to design primers amplifying a 300 – 600 bp fragment flanking the putative mutation, or part of the mutation for large LOH events. We then amplified the targeted fragment in the line housing the mutation and in another, randomly selected line using standard PCR protocols and conditions. The resulting PCR products were sequenced at Genome Quebec. Chromatograms were then inspected in MEGA v5.2 (Tamura *et al.* 2011) to verify or reject putative mutations.

### Genomic analyses - Statistical analyses

To calculate rates of SNM and LOH events per base pair per generation, we determined the number of sites at which mutations could have been called on a per line basis. For SNMs this was sites with at least six reads with mapping quality > 20 and base quality > 10 for that line and at least one read with such properties in all other lines. For LOH events, the same requirements applied, but additionally sites had to occur in a stretch of such sites at least 200 bases long interrupted by no more than six bases not meeting these requirements. Gaps of up to six bases were allowed to account for small INDELs between our ancestor and the reference genome. Rates were not calculated for INDELs or MNPs due to the small number of these mutations. For SNMs, we used one-way ANOVA to test if per base pair per generation mutation rates within lines differed across metal treatments. We used chi-squared tests to test if the 6 possible types of SNMs occurred at equal frequency and if the relative frequencies of each differed across metal treatments. For this analysis we corrected observed counts for genome-wide AT content. For LOH events, we used either one-way ANOVA (where assumptions of normality were met) or the non-parametric Kruskal-Wallis equivalent to test if metal treatment affected the per line number of LOH events per generation, the average LOH event size per line, or the number of basepairs effected per line per generation by LOH.

### Data availability

All genome sequence data have been deposited in the Sequence Read Archive (SRA) under accession PRJNA341529. Data from the life-history assays are provided in Supplementary File S2. Supplemental material available at Figshare: https://doi.org/10.25387/g3.7195802.

## Results

### Effects of mutation accumulation on intrinsic rate of increase and on body size at maturity

**Figure 1:**
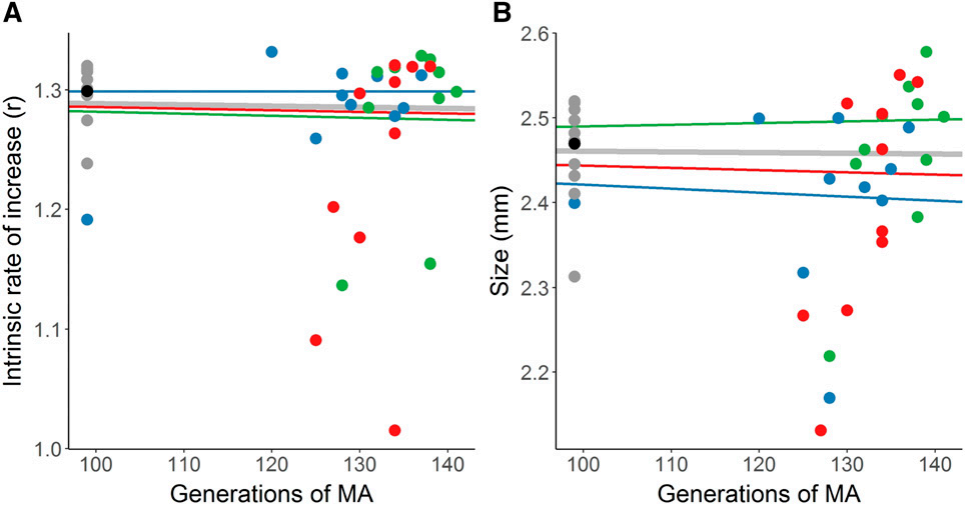
Effects of mutation accumulation on fitness-relevant traits. (A) Intrinsic rate of increase (r, unitless) and (B) body size as a function of the number of generations of mutation accumulation (MA) for *Daphnia pulex* mutation-accumulation lines exposed to metal-free conditions (blue) or to low-levels of copper (green), or nickel and copper (red). Gray points are lines from a population experiencing selection (arbitrarily shown at generation 99) and the black point is the mean of these values, taken as the pre-mutation accumulation value. Solid lines are the weighted-least squares line of best fit across (gray) or within (colors) treatments. n = 10 per treatment.

For intrinsic rate of increase (r), there was no significant effect of metal treatment on mutational bias (ΔM) (F_2, 24_ = 2.05, *P* = 0.15, Figure 1A, Table 1), and ΔM across all treatments was not significantly different from zero at -0.0079 ± 0.0062 (standard error) % per generation (t_29_ = 1.29, *P* = 0.21). There was no significant difference in between line variance among treatments (K^2^_2_ = 1.43, *P* = 0.49), with an overall coefficient of mutational variance (CV*_m_*) across all treatments of 6.87% or 0.052% per generation, and an overall mutation heritability (V_M_/V_E_) of 1.70% (Table 1). Within each treatment, except for copper, there was a significant effect of line on r (Control, F_9,43_ = 2.32, *P* = 0.03; Cu, F_9,35_ = 1.20, *P* = 0.33; Ni+Cu, F_9.36_ = 4.49, *P* < 0.001), *i.e.*, lines had significantly diverged from each other within that treatment. Tukey post-hoc analyses revealed significant differences were limited to one (Control; C012) or two (Ni+Cu; C352, C355) lines that differed from the remaining lines (Figure 1). Across all lines, rate of change in r was not significantly related to generation number (F_1,27_ = 1.531, *P* = 0.23, one outlier with 21 MA generations fewer than the closest line removed, Figure 1A).

For body size, there was a significant effect of metal treatment on ΔM (F_2,24_ = 7.97, *P* = 0.002, Figure 1B, Table 1). Pairwise comparisons showed ΔM was more negative in metal-free lines than in Ni+Cu lines (F_1,16_ = 12.36, *P* = 0.003), with other comparisons nonsignificant (F_1,16_ ≤ 2.31, *P* ≥ 0.15). However, in no treatment was ΔM significantly different from zero (t_9_ ≤ 1.94, *P* ≥ 0.08), nor was ΔM across all treatments significantly different from zero at -0.0035 ± 0.0062% per generation (t_29_ = 0.57, *P* = 0.56). There was no significant difference in between line variance among treatments (K^2^_2_ = 1.48, *P* = 0.48), with an overall coefficient of mutational variance (CV*_m_*) across all treatments of 9.81% or 0.075% per generation, and an overall mutation heritability (V_M_/V_E_) of 2.75% (Table 1). For each treatment except for copper, there was a significant effect of line on body size (Control, F_9,48_ = 2.95, *P* = 0.007; Cu, F_9,34_ = 1.30, *P* = 0.27; Ni+Cu, F_9.37_ = 8.78, *P* < 0.001), *i.e.*, lines had significantly diverged from each other within that treatment. Tukey post-hoc analyses revealed significant differences were limited to one (Control; C012) or two (Ni+Cu; C352, C358) lines that differed from the remaining lines (Figure 1). Across all lines, rate of change in body size was significantly positively related to generation number (F_1,27_ = 10.2, *P* = 0.004, r^2^ = 0.27, one outlier with 21 MA generations fewer than the closest line removed). Additionally, rate of decline in r was positively correlated with rate of decline in body size across all lines (Pearson correlation, r = 0.56, t_23_ = 3.20, *P* = 0.004). Across all treatments, broad-sense heritabilities were 0.69 and 0.78 for r and for body size respectively, indicating unaccounted-for environmental variations contributed to 31% and 22% of variation among individual sublines.

### Genomic analyses

We obtained a total of 1.05 billion reads across all lines of which approximately 85% mapped to the reference genome. This resulted in an average genome-wide coverage of 12.4 × per line (Table S1). After quality control and filtering, we retained an average of 72,163,836 sites (40.10% of the reference genome, range: 59,858,741 – 78,153,461) callable for SNM, MNPs and small INDELs per line and an average of 44,443,224 sites (22.53% of the reference genome, range: 27,518,873 – 52,538,839) callable for LOH events per line. Within SNM-callable sites, the recovered genome had an AT content of 58.18%, which is very similar to the full reference genome content of 57.43%. Across all lines, we identified a total of 644 SNM, 16 small INDELs and 67 LOH events (Tables S4 - S7). Our validations showed the bioinformatic pipeline used produced low false-positive rates: LOH events – 11 of 12 validated, INDELs – 5 of 5 validated, SNM - 25 of 26 validated (Table S3).

### Genomic analyses - SNMs

**Figure 2:**
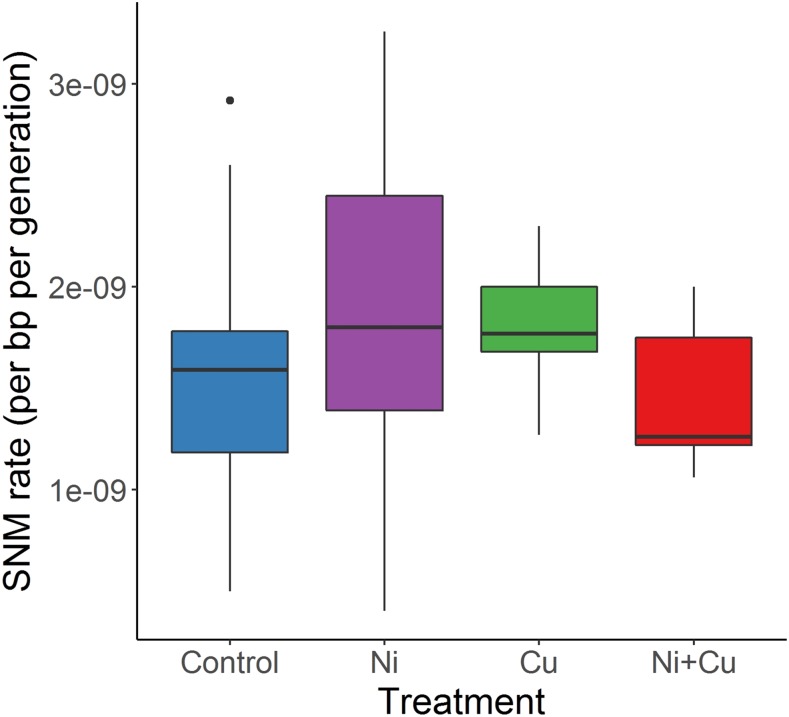
Single nucleotide mutation (SNM) rates in *Daphnia pulex* mutation-accumulation lines. Lines were exposed to metal-free (Control) conditions or to low-levels of nickel (Ni), copper (Cu), or nickel and copper (Ni+Cu). n = 28 for Control and 9 for other treatments.

**Figure 3:**
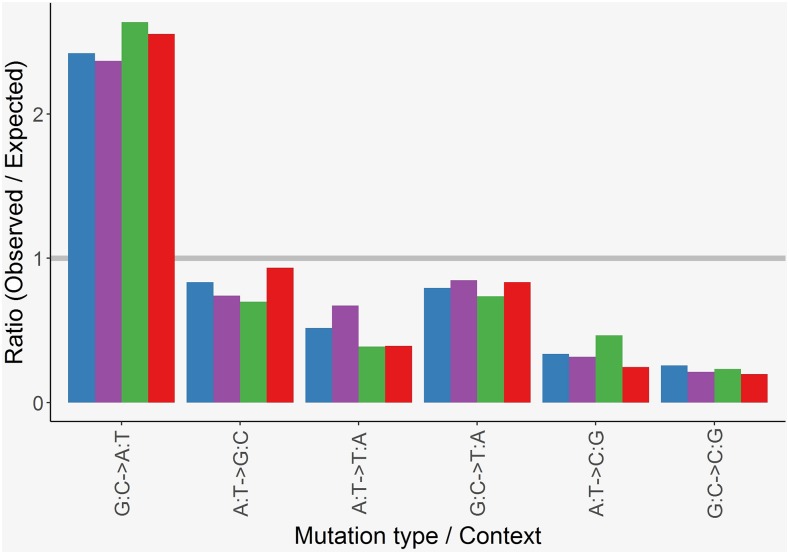
Single nucleotide mutation bias under mutation accumulation. Ratios between observe and expected counts of each of the six base substitutions for *Daphnia pulex* mutation-accumulation lines exposed to metal-free (control) conditions (blue) and to low-levels of nickel (purple), copper (green) or nickel and copper (red), pooled across lines within treatments. Expected values assume each substitution occurs at equal frequencies and correct for the unequal AT:GC content of the genome.

Overall, there was no evidence that metal treatment affected processes producing SNMs. There was no significant effect of metal treatment on the number of SNMs per bp per generation (one-way ANOVA, F_3,51_ = 0.994, *P* = 0.40, Figure 2, Table S4). The 644 SNMs detected across all lines corresponds to an overall SNM rate of 1.61 (± 0.08, standard error) * 10^−9^ bp/gen, with 95% confidence intervals of 1.46 – 1.77 * 10^−9^ bp/gen. Within SNMs, the six possible mutation types occurred at significantly different frequencies (χ^2^_9_ = 1579.9, *P* < 0.001, Figure 3), but the pattern was not significantly different across metal treatments (χ^2^_15_ = 6.55, *P* = 0.97, Figure 3). A total of 24 or 3.7% of all SNMs occurred in 10 MNMs, the percentage of which was unaffected by treatment (χ^2^_3_ = 5.22, *P* = 0.16). SNM rate was not linearly related to generation number across all lines (F_1,53_ = 0.024, *P* = 0.88).

### Genomic analyses - LOH events

**Figure 4:**
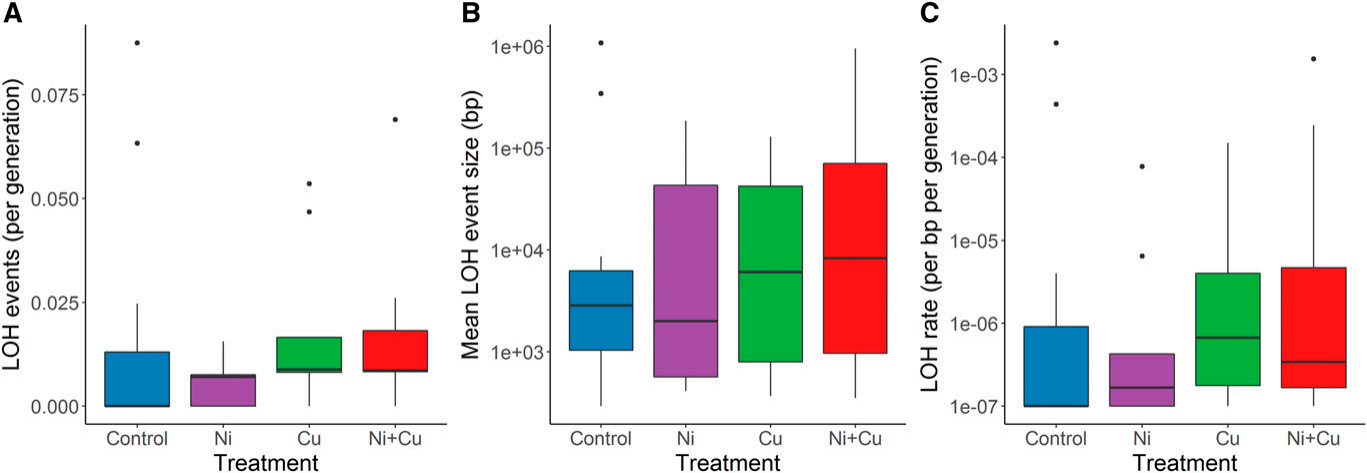
Loss of heterozygosity (LOH) under mutation accumulation. (A) Number of loss-of-heterozygosity events per generation, (B) mean LOH event size (log-scale), and (C) LOH rate (log-scale) in *Daphnia pulex* mutation-accumulation lines exposed to metal-free (control) conditions and to low-levels of nickel (Ni), copper (Cu), or nickel and copper (Ni+Cu). A small value (10^−7^) was added to each value in C to allow plotting on a log-scale as there were many values of zero. n = 28 for Control and 9 for other treatments.

Overall, there was no evidence metal treatment effected LOH mutational processes. There was no significant effect of metal treatment on the number of LOH events per generation (Kruskal-Wallis non-parametric ANOVA, χ^2^_3_ = 3.26, *P* = 0.35, Figure 4A), on the average LOH event size (one-way ANOVA, F_3,27_ = 0.13, *P* = 0.94, Figure 4B), or on the number of basepairs affected by LOH events per bp per generation (Kruskal-Wallis non-parametric ANOVA, χ^2^_3_ = 4.37, *P* = 0.22, Figure 4C). This was also true considering only gene conversion events (χ^2^_3_ = 1.83, *P* = 0.61; χ^2^_3_ = 2.75, *P* = 0.43 and χ^2^_3_ = 2.65 *P* = 0.45 respectively; all Kruskal-Wallis non-parametric ANOVAs, Figure S1A,B,C) and considering only hemizygous deletion events (χ^2^_3_ =2.74, *P* = 0.43; χ^2^_3_ =5.64, *P* = 0.13 and χ^2^_3_ = 4.16, *P* = 0.25 respectively; all Kruskal-Wallis non-parametric ANOVAs, Figure S1D,E,F). The 67 LOH events detected ranged in size from 229 bp to 7,349,102 bp (Table S7) and correspond to rates of 0.012 (± 0.019, standard deviation) events per generation, 3,755 (± 15301) affected bp per generation and 6.93 * 10^−5^ (± 2.88 * 10^−4^) per bp per generation. The large standard deviations relative to means for each of these measures reflects the very uneven distributions of LOH events across lines and of event sizes (Figure 4); individual lines had between 0 and 8 LOH events with 24 of 55 lines having no LOH events. Curiously, the three largest LOH events (1,718,070 bp, 7,336,814 bp, 7,349,102 bp) were all gene-conversion like events occurring in three separate MA lines and that overlapped each other on chromosome 11 (Table S7). In two cases, these MA lines were homozygous for one of the ancestral alleles, while the other MA line was homozygous for the other ancestral allele; read depth data supported diploidy in each case rather than hemizygosity (Table S7). LOH rate was not linearly related to generation number across all lines (F_1,53_ = 0.248, *P* = 0.62). Lines with a greater SNM rate were not likely to have more basepairs affected by LOH events on a per generation basis (Spearman rank-order regression, S = 29111, *P* = 0.72).

## Discussions

This study investigated the effects of chronic stress in the form of copper and nickel exposure on mutational processes in the model organism *Daphnia pulex* at phenotypic and genomic levels by using a combination of mutation accumulation, whole genome sequencing, and life-history assays. Our results provide no evidence that metal exposure affected rates for either single nucleotide mutations (SNMs) or loss-of-heterozygosity (LOH) events and, at most, very weak evidence of affecting rates of mutations for fitness relevant traits. We can reject the possibility *Daphnia* were not sufficiently stressed to invoke genetic responses in this experiment as we have previously shown rates of large-scale deletions and duplications are elevated fourfold under metal exposure in this experiment (Chain *et al.* 2018).

### Stress contributes negligibly to fitness decline

Theory predicts that for quantitative traits, such as intrinsic rate of increase and body size at maturity, mutation accumulation will result in increased variance among lines, due to unique sets of mutations arising in each line, and a directional change in mean trait value if on average mutations have a directional effect (Lynch and Walsh 1998; Halligan and Keightley 2009). These predictions are routinely validated in MA experiments (Mukai 1964; Lynch 1985; Vassilieva and Lynch 1999). If exposure to a putative stressor increases the rate at which these phenomena occur an increased mutation rate can be inferred (Matsuba *et al.* 2013). Although we found no statistical evidence for either phenomenon, trends were in the direction predicted if metal-exposure was mutagenic for intrinsic rate of increase; both the rate of fitness decline and the coefficient of mutational variance were higher in metal-exposed lines than in control lines. Thus, there is some evidence for effects of metals on the rate of fitness-affecting mutations; however, the effects detected here must be minor.

Averaging across treatments, our estimate of a decline of 0.008% per generation for intrinsic rate of increase, which was not significantly different from zero, is the lowest reported for multicellular organisms. Previous estimates range from 0.03% per generation in *Drosophila melanogaster* (Caballero *et al.* 2002) to 0.31% per generation in *Caenorhabditis briggsae* (Baer *et al.* 2005). The wide standard errors for our estimate (0.006%) means our estimate overlaps the lower end of these other estimates, and it is unclear if mutational decay is indeed slower in *Daphnia*. This might not be surprising given *Daphnia* is unique among multicellular animals that have been subjected to MA in that it is maintained in a heterozygous state throughout the experiment due to propagation via parthenogenetic reproduction (Halligan and Keightley 2009). Thus, most classes of novel mutations are assayed in a heterozygous state. If novel deleterious mutations are on average recessive or partially so, as suggested by studies of induced mutations in *Caenorhabditis elegans* (Peters *et al.* 2003), a more gradual fitness loss would be expected. Our findings that the mean change in body size was not significantly different from zero across treatments further points to such phenomena. However, if body size was previously under stabilizing rather than directional selection (De Meester and Weider 1999), no change in mean might be expected under mutation (Houle *et al.* 1992; Houle *et al.* 1996), and the importance of this finding is unclear. Additionally, it is possible our non-MA population evolved a smaller body size throughout the course of the experiment due to domestication selection, masking the usually observed decline in body size in *D. pulex* MA experiments (Lynch *et al.* 1998). Although the MA design aims to minimize within-line selection, among-line selection for high fitness is still possible if lines that evolve the lowest fitness are most likely to go extinct, *i.e.*, - a potential bias against observing fitness declines exists, which could also partially explain our findings. This is however true of all MA experiments, and it is unclear that ours should be more effected than other studies; 9.3% of all lines that could have been assayed for fitness were lost to extinction in our study, lower than previous studies reporting significant fitness loss, *e.g.*, – a 12% loss of initial lines over 104 generations in *Drosophila melanogaster* in Fernández and López-Fanjul (1996) and a 27% loss of initial lines over 214 generations in *C. elegans* in Vassilieva *et al.* (2000). While it is true our study is unique in applying a chronic, mild stress during propagation, any additional selective effects due to this would presumably be reflected in greater line extinction rates. The extent to which within-line selection may have been affected is harder to determine; the proportion of times a backup was employed due to the death of the main individual partially captures this information (4.8% of all transfers in our study) but is rarely reported in MA studies, limiting comparison.

Previous mutation accumulation studies in *Daphnia* have, however, found significant declines in fitness relevant traits over fewer generations than used here (Lynch *et al.* 1998; Latta *et al.* 2013), indicating masking of recessive novel mutations is unlikely to offer a complete explanation. Notably, one of these studies used twenty different genotypes of *Daphnia pulicaria* and found rates of mutational decay of fitness to be strongly genotype-dependent (Latta *et al.* 2013). Further, certain genotypes in that study showed no change in measured traits over up to 65 generations, *i.e.*, - they were mutationally robust, exhibiting stable phenotypes despite genetic changes (Masel and Siegal 2009). Given the per-generation genomic mutation rates for *Daphnia* reported here and elsewhere (Keith *et al.* 2016; Flynn *et al.* 2017), it is unlikely that MA lines of these genotypes were free of novel mutations after 65 generations. Other studies have shown the degree of robustness to vary across genotypes within species (Fu *et al.* 2009). Given that mutationally robust genotypes appear to exist in the closely related sister species to that used here, and that we know each MA line in our study has accumulated at least several SNMs but overall changes in measured phenotypic traits were small, it seems likely the lineage used here may be generally mutationally robust. Although we did find evidence that lines within treatments had diverged over the course of this experiment, this was restricted to one or two lines per treatment which had changed markedly from the initial phenotype. This is consistent with general mutational robustness and rare mutations of sufficient effect size to overcome robustness (Davies *et al.* 1999), or mutations that otherwise interfere with the molecular mechanisms producing robustness (Masel and Siegal 2009). The only common feature of lines with rapid declines in r were high CNV rates, which is consistent with the potential of such mutations to have large fitness effects by disrupting gene-dose relationships (Katju and Bergthorsson 2013). However, given high CNV rates were not *per se* predictive of rapid r declines, the nature of genetic regions effected by specific CNVs is likely the most important factor, with lines with more CNVs being more likely, but not guaranteed, to host strongly fitness-effecting mutations.

The curious finding that rate of change in mean body size was related to number of generations under MA deserves some explanation. Most likely, some lines acquired mutations that cause both a reduced rate of generational advance in the MA experiment (late first reproduction, poor survival) and a smaller body size. Such mutational co-variance is common in *Daphnia* (Lynch 1985), and indeed age at first reproduction and body size were negatively related in this study (Table S2).

### No effects of stress on SNM and LOH producing processes

Our finding of no change to the SNM rate or spectrum under stress is consistent with the only previous MA study involving multicellular animals able to assess such changes, that of Sharp and Agrawal (2016) in *Drosophila melanogaster*, which used MA lines of high and low genetic quality, despite the fact we used an externally applied stress. However, our results are inconsistent with a study in *Arabidopsis thaliana* which found SNM rates were higher and more transition-biased during MA in saline soils, a common plant stressor, compared to control conditions (Jiang *et al.* 2014). It should be noted that at the level of salinity used in Jiang *et al.* (2014) resulted in pronounced growth retardation indicating the level of stress applied is likely to be much higher than that used here and in Sharp and Agrawal (2016), where the most loaded genotype had a ∼11% reduction in mass compared to the wild-type control.

Failure to find modified SNM rates of spectra under copper and nickel exposure is not surprising given *in vitro* interactions between DNA and these metals are stereotyped as causing double stranded breaks (DSBs) (Guecheva *et al.* 2001; Valko *et al.* 2006) via the generation of reactive oxygen species (ROS) (Tkeshelashvili *et al.* 1991; Beyersmann and Hartwig 2008). DSBs are more typically incorrectly repaired as LOH or copy number variants (CNVs) than SNMs (Lieber 2010). ROS production however can also lead to nucleotide modifications which when incorrectly repaired result in C->T transitions (Tkeshelashvili *et al.* 1991). Such mutations are the most common SNM observed in our study, but this is true for all treatments including those without metals. Rates for C->T rates in treatments containing Nickel are slightly higher than other treatments, which may reflect that cellular free-copper levels are typically more strongly regulated than cellular free-nickel levels in animals (Beyersmann and Hartwig 2008), but any effect if present is small. Non-MA studies have shown certain stressors can modify SNM rates and spectra in multicellular animals, *e.g.*, the application of certain antibiotics resulted in increased SNM rates in *C. elegans* (Meier *et al.* 2014), but the finding of no effects in this study does indicate modified SNM rates or spectra are unlikely to be a ubiquitous stress response over multiple generations.

Given the above-described mechanism of copper and nickel associated DNA damage, it is surprising that no effects of metal exposure on LOH generating processes were detected. This is especially true given that CNV deletion and duplication rates were raised fourfold in nickel and copper lines compared to no-metal MA lines in the same set of MA lines described here (Chain *et al.* 2018). It is of course also possible we simply lacked statistical power to detect any effects; between line variability with regard to LOH events was extreme; individual lines had up to 8 LOH events while 24 of 55 lines had no events, and lines had between 0 and 7,609,617 bp affected. That the three largest LOH events should overlap is remarkable, with only a 0.7% chance of them occurring on the same chromosome (1 * 1/12 * 1/12 - *D. pulex* has 12 chromosomes, assuming these events occur in different lines as was the case here), let alone overlapping. Curiously, the one MA line containing one of these mutations that was subjected to fitness assay did not display markedly reduced r or body size. The other two lines, however, went extinct prior to fitness assays, indicating such mutations are probably, on balance, deleterious. Because of the multiscaffold nature of such mutations we cannot identify conversion initiation points, however remarkably localized chromosome fragility is implied.

Given that modifications to the mutation spectrum were apparently highly specific and in manners consistent with the know mechanisms of interaction of copper and nickel with DNA (Beyersmann and Hartwig 2008), stressor-specific mutagenic effects are likely the most parsimonious explanation for our findings. This is also supported by our failure to detect an increase in the percentage of SNMs contained in MNMs under copper and nickel exposure, as this is a typical signature of the stress-general low-fidelity polymerases used by some unicellular organisms (Fitzgerald *et al.* 2017); expression of these polymerases could be quantified directly in future studies where effects are detected. At higher metal concentrations, more general stress responses may become important given the potential for these metals to cause physiological as well as genomic stress (Fernandez-Gonzalez *et al.* 2011).

### Evolutionary implications

Taken together, the results of this study and a previous study demonstrating elevated large-scale deletion and duplications under metal stress in the same lines (Chain *et al.* 2018), indicate that chronic exposure to mild copper and nickel stress does result in increased mutation rates, but that these increases are restricted to specific mutations classes. As fitness consequences are apparently limited, or at least masked by general mutational robustness is the lineage studied here, this increased rate is unlikely to contribute meaningfully to the evolutionary response of populations exposed to such stress. It is worth reinforcing that we used mild concentrations of copper and nickel without detectable effects on survival or fitness. It is likely that exposure to high levels of metals would result in strong mutagenesis that would lead to direct fitness consequences (Beyersmann and Hartwig 2008). The relative importance of mutagenic and of direct fitness effects on population persistence and evolution under such exposures is unclear but probably depends on the magnitude of direct fitness consequences, magnitude of increased mutation input, population size and duration of exposure (Lynch and Gabriel 1990).

More broadly, these results suggest mutation rates may be less environmentally responsive in multicellular organisms than in unicellular organisms. This may not be surprising given the substantial reasons, relating the higher initial mutation rates and smaller effective population sizes (Kimura 1967; Lynch 2010). Further, multicellularity may reduce the effective intensity of a given stress relative to that experienced by unicellular organisms by other mechanisms, such as by reduced surface area for metal uptake. Although some studies have demonstrated elevated mutation rates under stress in multicellular organism (*e.g.*, Matsuba *et al.* 2013; Sharp and Agrawal 2016), such results are not ubiquitous (*e.g.*, Mackenzie *et al.* 2005) and the wide variety of stressors, stress intensities and study organisms used makes comparisons difficult. This is further complicated by the fact that some genotypes are apparently mutationally-robust even in the face of stress, as shown here. More research is clearly required before we can begin to determine general patterns in the environmental response of mutation rates in multicellular organisms.
